# Experiences of breastfeeding during COVID‐19: Lessons for future practical and emotional support

**DOI:** 10.1111/mcn.13088

**Published:** 2020-09-23

**Authors:** Amy Brown, Natalie Shenker

**Affiliations:** ^1^ Department of Public Health, Policy and Social Sciences Swansea University Swansea UK; ^2^ Centre for Lactation, Infant Feeding and Translation Swansea University Swansea UK; ^3^ Department of Surgery and Cancer Imperial College London London UK

**Keywords:** breastfeeding, breastfeeding support, COVID‐19, formula feeding, lockdown, maternal mental health

## Abstract

The COVID‐19 pandemic and subsequent lockdown and social distancing led to changes to breastfeeding support available to women in the United Kingdom. Face‐to‐face professional support was reduced, and face‐to‐face peer support was cancelled. Anecdotal media accounts highlighted practices separating some mothers and babies in hospitals, alongside inaccurate stories of the safety of breastfeeding circulating. Meanwhile, new families were confined to their homes, separated from families and support networks. Given that we know breastfeeding is best supported by practices that keep mother and baby together, high‐quality professional and peer‐to‐peer support, and positive maternal well‐being, it is important to understand the impact of the pandemic upon the ability to breastfeed. To explore this, we conducted an online survey with 1219 breastfeeding mothers in the United Kingdom with a baby 0–12 months old to understand the impact of the pandemic upon breastfeeding duration, experiences and support. The results highlighted two very different experiences: 41.8% of mothers felt that breastfeeding was protected due to lockdown, but 27.0% of mothers struggled to get support and had numerous barriers stemming from lockdown with some stopped breastfeeding before they were ready. Mothers with a lower education, with more challenging living circumstances and from Black and minority ethnic backgrounds were more likely to find the impact of lockdown challenging and stop breastfeeding. The findings are vital in understanding how we now support those women who may be grieving their loss of breastfeeding and are affected by their negative experiences and how we can learn from those with a positive experience to make sure all breastfeeding women are better supported if similar future events arise.

**Key messages**

- The COVID‐19 pandemic has affected women's breastfeeding experiences in the United Kingdom. For some, this was positive because of increased time at home, less pressure and fewer visitors.
- Others reported more challenging experiences, struggling to get support, worrying about the safety of feeding and feeling isolated. These women were more likely to stop breastfeeding before they were ready, directly blaming the impact of the pandemic.
- Women who had a more difficult breastfeeding experience lived in more challenging circumstances. BAME women and those with a lower education were more likely to be represented in this group.

## 1 BACKGROUND

The COVID‐19 pandemic has challenged our approach to almost every aspect of life (Kickbusch et al., 2020). Since the onset of the pandemic, over five million cases of COVID‐19 have occurred globally with 500,000 deaths (World Health Organization [WHO], 2020a), and numerous countries, including the United Kingdom, have endured often prolonged lockdown measures to encourage social distancing and limit the spread of the virus (Davies et al., 2020). In the United Kingdom, a lockdown was imposed on the evening of 23 March, with the majority of public places closing (apart from essential stores), travel restrictions and meeting with those from other households limited, apart from for caring or work‐based reasons.

One group that was particularly affected by lockdown measures was new parents. Many had to adapt rapidly to changing and uncertain circumstances, with scarce information and frequently mixed messages from major public health bodies (Renfrew, Cheyne, Craig, et al., 2020). Changes to breastfeeding support were part of this. We know that breastfeeding works best when women receive high‐quality support (McFadden et al., 2017), including promotion immediately post birth of skin‐to‐skin contact, mother and baby remaining together and support to initiate breastfeeding as soon as possible after birth (Gavine, McFadden, MacGillivray, & Renfrew, 2017). Thereafter, receiving continued support in the community is particularly important to breastfeeding success (Pérez‐Escamilla, Martinez, & Segura‐Pérez, 2016).

However, many of these aspects of gold standard care have been affected by the pandemic. As a novel pathogen, no data were initially available on whether SARS‐CoV‐2 could be vertically transmitted from mother to infant in utero or postnatally, through direct respiratory inhalation or breastfeeding (Juan et al., 2020). These concerns led to anecdotal reports in the local and international news and on globally shared social media posts of mothers giving birth without partner or doula support, being separated from their infant after birth or being told that breastfeeding was not safe (Guardian, 2020; Pramono, Dahlen, Desborough, & Smith, 2020). This was despite action in the United Kingdom from breastfeeding and public health organisations to emphasise the safety and importance of continued breastfeeding (UNICEF UK, 2020).

Meanwhile, in the community, many health visitors were redeployed into nursing roles, face‐to‐face breastfeeding support groups were moved online or abandoned and antenatal care was delivered largely online. More broadly, there was much fear in communities around infection spread, and social distancing requirements removed family support and contact for many new parents.

At the time of writing, no published peer‐reviewed data exists on the impact of COVID‐19 on breastfeeding outcomes and decisions around infant feeding in the United Kingdom. This mixed‐methods online survey study examined the experiences of over 1200 women with infants under the age of 1 year to understand how the COVID‐19 pandemic affected their infant feeding attitudes, choices and outcomes. A greater understanding of these impacts will guide practice and policy with regards to supporting pregnant and new mothers.

## 2 METHODOLOGY

### 2.1 Participants

Full ethical permission was gained from a University Research ethics committee. Participants were provided with participant information sheets describing the content of the questionnaire and responded to consent questions before the full questionnaire opened. A short debrief was included with further details of where to seek support with infant feeding, mental health or further concerns about themselves or their infant.

UK mothers who had breastfed their baby aged 0–12 months at least once during the COVID‐19 pandemic (identified as since 1 March 2020) completed a questionnaire. It is recognised that what constitutes the start of the pandemic in the United Kingdom is debatable. However, this date provided an easily memorable cut‐off date for recall purposes. Different areas in the United Kingdom started experiencing changes to services during February and March, with internet articles circulating around infant feeding during these months. Therefore, the effects of the pandemic were likely to have started affecting maternal well‐being and decisions from around this date.

We chose an age range of 0–12 months for this analysis as we wanted to explore the impact of the pandemic upon breastfeeding experiences and cessation. Most breastfeeding complications, need for greater support and decisions to stop prematurely happen in the earlier months of feeding, although it is recognised that breastfeeding continues for longer. We wanted to explore whether only mothers of a younger infant attempting to establish breastfeeding would be affected or whether impacts would be seen for older, established breastfeeding dyads too. We therefore examined outcomes for mothers dependent on whether their baby was born before or during the pandemic using the cut‐off date of 1 March 2020. Using this cut‐off date, rather than the date of lockdown, although approximate, allowed us to include more mothers whose very early feeding experiences in the first few weeks may have been affected by lockdown, despite giving birth before it occurred. It is recognised that the exploration of breastfeeding experiences for these two groups will be confounded by the younger age of the infant amongst those giving birth after the pandemic started.

### 2.2 Measures

Participants completed a questionnaire containing both closed and open questions, hosted online by Qualtrics. The questionnaire included

- Demographic details (maternal age, education, ethnicity, parity, live‐in partner and infant age)
- Details of breastfeeding duration and formula use
- Readiness and reasons for stopping breastfeeding
- Information encountered around the safety of COVID‐19 and breastfeeding
- Perceptions of the impact of COVID‐19 and lockdown upon breastfeeding experience
- Individual circumstances during the pandemic (e.g., internet access, financial difficulties and housing set up)

### 2.3 Procedure

Data were collected for 4 weeks during May–June 2020. Adverts were placed on social media with encouragement for breastfeeding organisations to share the post. During the study, our post was shared over 500 times across social media platforms. Each post contained brief details of the study and inclusion criteria with a link to the questionnaire. Interested participants could click on the link and the participant information sheet and consent questions loaded. Once completed, a debrief statement was given, explaining the study, thanking them for participation and giving them contact details for support organisations if needed.

### 2.4 Data analysis

Quantitative data were analysed using SPSS version 22. Descriptive statistics explored breastfeeding duration, feeding experiences and reasons for stopping. Participants were grouped into dichotomous variables for demographic measures university‐level/not university‐level education, White/Black and minority wthnic (BAME) ethnicity, primiparous/multiparous and live‐in partner/not live‐in partner. Feeding data were used to calculate current feeding methods (exclusive breastfeeding, mixed feeding and exclusive formula feeding) with any breastfeeding (exclusive or mixed) used to determine continued breastfeeding at the time of the survey (yes/no).

*t* tests, chi‐square and Spearman's rho correlations explored associations between breastfeeding continuation/cessation and misinformation, fears and individual circumstances. Chi‐square and *t* tests explored associations between infant feeding experiences and demographic background. Multivariable logistic regression models were used to explore the association between individual living circumstances, maternal education and breastfeeding experience. Differences in outcomes and experiences were also compared for mothers giving birth before or after the pandemic using chi‐square.

A thematic analysis was conducted on qualitative data from the open‐ended boxes. Responses were read and reread to identify smaller themes, and then we group these smaller subthemes into larger subthemes. Initial coding was completed by one researcher, with a second reviewing themes and subthemes. Where disagreement occurred, themes were discussed until agreed (Braun & Clarke, 2014).

### 2.5 Ethical considerations

Ethical approval was granted by Swansea University College of Human and Health Sciences research ethics committee.

## 3 RESULTS

One thousand two hundred nineteen participants completed the questionnaire with a mean age of 30.92 (SD: 6.119; range 18–46). The mean age of infants was 13.24 weeks (SD: 13.19; range 1–52). Four hundred ninety‐five (40.6%) gave birth before the pandemic and 724 (59.4%) during it.

At the time of survey completion, 715 (58.6%) participants were breastfeeding exclusively, 274 (22.5%) mixed feeding and 230 (18.9%) had stopped breastfeeding. Mean infant age at the introduction of infant formula was 2.79 weeks (SD: 4.12; range 1–36 weeks) and 3.15 weeks for breastfeeding cessation (SD: 3.81; range 1–38). Of those who stopped breastfeeding during the pandemic, 91.3% of participants had given birth during the pandemic compared with 8.7% who gave birth before. The majority of these infants (82.5%) were age 6 weeks or younger. Further demographic details can be found in Table 1.

**TABLE 1:** Participant demographic background

Indicator	Group	*N*	%
Age	≤19	35	2.9
	20–24	114	9.0
	25–29	304	24.9
	30–34	456	37.4
	≥35	310	25.8
Education	School	206	19.5
	College	324	26.6
	Higher	379	31.1
	Postgraduate	310	25.4
Ethnicity	White	1118	91.7
	Gypsy/traveller	5	0.4
	Asian or Asian British (Bangladeshi, Indian and Pakistani)	41	3.3
	Asian or Asian British (Chinese)	23	1.9
	Black or Black British	14	1.1
	Mixed or multiple	14	1.1
	Prefer not to say	4	0.3
Parity	First baby	713	58.6
	Second or more	506	41.4
Partner at home	Yes	1162	95.3
	No	57	4.7
Country	England	896	73.5
	Scotland	131	10.7
	Wales	89	7.3
	Northern Ireland	71	5.8
	Ireland	32	2.6

Mothers who were still breastfeeding were more likely to have a degree or postgraduate qualification compared with those no longer breastfeeding (*χ* ^2^ = 60.935, *P* = 0.000), living with a partner (*χ* ^2^ = 8.665, *P* = 0.005) and be multiparous (*χ* ^2^ = 14.456, *P* = 0.000). BAME mothers were less likely to still be breastfeeding compared with White mothers (*χ* ^2^ = 10.770, *P* = 0.001).

### 3.1 Feeding intentions versus reality

Of those participants who had stopped breastfeeding, only 13.5% described themselves as ready to do so. Little difference was seen in readiness to stop between those who gave birth before (14.3%) or after (13.4%) the pandemic. When asked whether they stopped sooner, later or when planned, just 4.7% stated they stopped when planned. Although 11.1% of the participants did not plan a duration, the majority (64.8%) had planned to feed for longer, although 19.4% breastfed longer than planned.

Meanwhile, 68.7% of the participants who had introduced formula stated they had never intended to do so, with 13.5% of the participants introducing it earlier than planned. Little difference was seen in never intending to introduce formula between those who gave birth before (70.6%) or after (67.7%) the pandemic. Conversely, 3.6% breastfed exclusively for longer than intended (3.8% who gave birth before and 3.5% who gave birth after the pandemic).

### 3.2 Reasons for breastfeeding cessation

Participants indicated how strongly they agreed with a series of reasons for stopping breastfeeding (*strongly agree*–*strongly disagree*, 5‐point Likert scale). The most common reason for cessation was insufficient professional support followed by physical issues such as difficulties with latch, exhaustion, insufficient milk and pain (Table 2). A multivariate analysis of covariance (MANCOVA) controlling for maternal age and education found no significant differences in reasons for stopping breastfeeding between those who gave birth before or during the pandemic.

**TABLE 2:** Participants who agreed with each reason for breastfeeding cessation, split by those who gave birth during or before the pandemic

	Whole sample		During pandemic		Before pandemic	
Reason for cessation	*N*	%	*N*	%	*N*	%
Insufficient professional support	182	79.1	167	79.9	15	71.4
Issues with latch	147	64.2	134	64.4	13	61.9
Exhaustion	128	56.1	115	55.6	13	61.9
Insufficient milk	111	48.3	103	49.3	8	39.0
Pain	106	46.1	97	46.4	9	42.9
To let other people feed the baby	71	37.0	65	31.1	6	28.5
Infant weight gain	84	36.5	117	56.0	10	47.6
Wanted to see how much baby was drinking	73	31.7	68	32.5	5	23.8
Partner attitude	61	26.5	56	26.7	5	23.8
Other responsibilities	53	23.0	48	23.0	5	23.8
Family attitude	52	22.6	48	23.0	4	19.0
Formula easier option	36	15.7	34	16.3	2	9.6
Medication	24	10.5	22	10.7	2	9.5
Embarrassment	18	8.7	17	8.2	1	4.8

In terms of how COVID‐19 had affected their decision to stop, 70.3% attributed a lack of face‐to‐face support, 20.9% worries about the safety of breastfeeding and 6.5% their symptoms of COVID‐19 to stopping breastfeeding. In terms of birth timing, 72.6% of mothers who gave birth during the pandemic attributed a lack of face‐to‐face support, 22.0% worries about the safety of breastfeeding and 12.5% their symptoms of COVID to stopping breastfeeding compared with 42.9%, 9.6% and 0%, respectively, of those who gave birth before it. The association between birth timing and a lack of face‐to‐face support was significant (*χ* ^*2*^ = 11.131, *P* = 0.025).

Mothers from BAME backgrounds were significantly more likely than White women to attribute a lack of face‐to‐face support to breastfeeding cessation (*t*(227) = 2.161, *P* = 0.032). Mothers without a university‐level education were more likely to have stopped over safety worries (*t*(227) = −2.044, *P* = 0.042). No differences were found for parity.

### 3.3 Safety fears

All participants were asked about their concerns around the safety of COVID‐19 and breastfeeding, including messages that they received from health professionals, social media and family and friends. Overall, 13.2% of mothers said that they worried about the safety of breastfeeding during COVID‐19, although the majority of that 13.2% stated that they worried at the start but were no longer worried now (80.3%). Meanwhile, 4.3% were told by a health professional that breastfeeding might not be safe during COVID‐19 and 3.3% that they would not be ‘allowed’ to breastfeed if they had symptoms. Additionally, 21.9% saw articles on social media that breastfeeding might not be safe, with 9.9% being given this opinion by friends and family. No differences were seen by maternal demographic background.

Significant associations were seen between being told breastfeeding may not be safe and current feeding group. Participants who had stopped breastfeeding were more likely to have been told breastfeeding was not safe by a health professional (*χ* ^2^ = 18.84, *P* = 0.000) or friends and family (*χ* ^2^ = 5.327, *P* = 0.011) or that breastfeeding would not be allowed with symptoms of COVID‐19 (*χ* ^2^ = 3.788, *P* = 0.047). No significant association was seen between current feeding and exposure to social media articles (*χ* ^2^ = 1.216, *P* = 0.155).

Anxieties over the pandemic may also have affected whether mothers contacted health professionals. When asked whether they contacted their health professional if they needed to, 48.6% stated they had no need. However, 16.4% stated they did not contact their health professional because of the pandemic, effectively 31.9% of those who needed support. Similar avoidance was found between those who gave birth during (30.5%) or before (34.4%) the pandemic. A significant association was found between not contacting a health professional and stopping breastfeeding (*χ* ^2^ = 21.388, *P* = 0.000).

Likewise, 58.8% stated they were concerned, or would be, if they needed to see a health professional face to face for breastfeeding complications. However, this was not significantly related to current feeding approach (*χ* ^2^ = 1.552, *P* = 0.460) and was at similar rates amongst those who gave birth during (58.2%) or before the pandemic (55.6%).

### 3.4 Impact of lockdown on immediate postnatal breastfeeding experience

Participants who had given birth after 1 March 2020 were asked to describe their postnatal feeding and care experiences. Overall, 7.8% stated they were not supported to have skin to skin, 4.6% were not encouraged to breastfeed as soon as possible after birth, 24.6% were not given information on expressing milk, and 21.2% stated they received no breastfeeding support in hospitals.

Participants who had a baby in neonatal intensive care unit (NICU) were asked whether they could visit their baby. Of the 103 mothers who did, 19.4% (*n* = 20) were told they could not visit their baby in NICU. Not being able to visit their baby in NICU was associated with no longer breastfeeding (*χ* ^2^ = 44.645, *P* = 0.000). At the time of survey completion, 80.0% who were told they could not visit their baby were no longer breastfeeding compared with 9.6% of those who could.

Participants who had symptoms of COVID‐19 (*n* = 25) were asked further questions. Of these participants, 16.0% were told they could not keep their baby with them after birth because of symptoms, but just 51.4% were told to wear a mask to feed their baby. Conversely, of the whole sample, 15.4% of participants were told to wear a mask to feed their baby when they did not have symptoms of COVID‐19.

### 3.5 Impact of lockdown upon breastfeeding support

Participants were asked whether they felt the lockdown overall had a positive or negative impact on their breastfeeding experience. Overall, 41.8% felt it was positive, 29.5% neutral and 27.0% negative. A further 1.7% were unsure of its impact. There was a strong significant association between perceptions and current feeding group (*χ* ^*2*^ = 247.362, *P* = 0.000). Whereas 48.8% of those still breastfeeding felt the experience had been positive, just 15.9% of those no longer breastfeeding felt this way. Perception was affected by the timing of birth: 36.2% of those who gave birth during the pandemic felt it was positive, 27.3% neutral, 34.8% negative and 1.7% unsure compared with 50.1% positive, 32.5% neutral, 10.7% negative and 1.8% unsure for those who gave birth before it. This association was significant (*χ* ^*2*^ = 71.526, *P* = 0.000) but will be confounded by the age of the infant.

The impact of lockdown was also linked to maternal demographic background. BAME women were significantly less likely to describe the impact as positive (*χ* ^2^ = 15.574, *P* = 0.000) compared with White women, whereas those with a university education were also more likely to describe it as positive than those without (*χ* ^2^ = 10.809, *P* = 0.004). No significant association was found for parity or living with a partner.

Using a 5‐point Likert scale, participants rated whether they felt they received enough practical and emotional support. Overall, 39.8% of participants felt they had enough practical support and 36.0% enough emotional support from health professionals. Mothers who were still breastfeeding were significantly more likely to state they had enough practical (*t*(1177) = 6.66, *P* = 0.000) and emotional (*t*(1177) = 7.198, *P* = 0.000) support.

Perceptions of practical (*χ* ^*2*^ = 22.629, *P* = 0.000) and emotional (*χ* ^*2*^ = 38.831, *P* = 0.000) support differed significantly by the timing of birth. For those who gave birth during the pandemic, 38.2% felt they had enough practical support and 34.4% enough emotional support compared with 42.3% and 38.8%, respectively, for those who gave birth before the pandemic.

Mothers from BAME backgrounds (*t*(1177) = −.203, *P* = 0.046) were significantly less likely to feel they had enough practical support. No other significant demographic differences were found in support for education, partner at home or parity.

A final support question asked via a 5‐point Likert scale whether participants felt they had more or less breastfeeding support during the lockdown. Overall, 4.0% felt they had more support, 22.3% about the same, 67.0% less support and 6.7% were unsure. For birth timing, 68.8% of those who gave birth during the pandemic felt they had less support compared with 67.8% who gave birth before it.

*t* tests found no significant differences in perception were seen between education, ethnicity or parity groups.However, there was a very strong association between perception of support and feeding group (*χ* ^2^ = 125.75, *P* = 0.000). All of those who perceived there to be more support were still breastfeeding. For perceiving less support, 89.3% of those no longer breastfeeding held this view compared with 63.2% of those still breastfeeding.

### 3.6 Impact of lockdown upon breastfeeding experience

In terms of specific impacts of the lockdown upon breastfeeding experience, such as having no visitors, or baby groups being cancelled, clear differences were seen between those who were still breastfeeding or not (Table 3). Some of each group found a more distant way of life positive, but those were still breastfeeding were much more likely to feel this way. However, many found this impact negative, with high proportions in the group who had stopped breastfeeding perceiving this lack of social and emotional support to have negatively impacted their breastfeeding experience.

**TABLE 3:** Perceived impact of lockdown consequences upon breastfeeding experience by current feeding method

	Positive impact %		Negative impact %		Significance
	Breastfeeding	Not breastfeeding	Breastfeeding	Not breastfeeding	
Having older children at home	30.4	13.3	24.3	42.1	*χ* ^2^ = 19.25, *P* = 0.001
Having fewer visitors in hospital	25.0	14.6	13.8	59.5	*χ* ^2^ = 162.32, *P* = 0.000
Having to stay home	20.9	4.9	50.8	81.4	*χ* ^2^ = 88.86, *P* = 0.000
Not having close family visit at home	14.5	5.4	52.1	87.8	*χ* ^2^ = 121.65, *P* = 0.000
Not having other visitors	32.6	11.0	36.4	75.8	*χ* ^2^ = 145.669, *P* = 0.000
Not being able to go to face‐to‐face peer support groups	1.2	0.0	72.8	92.8	*χ* ^2^ = 124.721, *P* = 0.000
Not being able to go to baby clinics	2.8	1.4	70.6	91.3	*χ* ^2^ = 125.751, *P* = 0.000

Differences in perceptions by birth timing were explored for those who gave birth before or during the pandemic (Table 4). Chi‐square found significant associations between experience and birth timing; mothers who gave birth during the pandemic consistently found impacts significantly more negative than those who had given birth before the pandemic.

**TABLE 4:** Perceived impact of lockdown consequences upon breastfeeding experience for those giving birth during or before the pandemic

	Positive impact %		Negative impact %		Significance
	During pandemic	Before pandemic	During pandemic	Before pandemic	
Having older children at home	27.1	29.3	27.2	26.5	*χ* ^2^ = 0.632, *P* = 0.959
Having to stay home	21.2	17.1	57.4	58.3	*χ* ^2^ = 10.54, *P* = 0.032
Not having close family visit at home	16.6	10.3	60.7	58.9	*χ* ^2^ = 13.59, *P* = 0.009
Not having other visitors	31.1	23.0	55.5	45.3	*χ* ^2^ = 13.93, *P* = 0.008
Not being able to go to face‐to‐face peer support groups	0.8	0.9	79.1	74.7	*χ* ^2^ = 19.62, *P* = 0.001
Not being able to go to baby clinics	2.4	77.9	2.6	71.0	*χ* ^2^ = 19.251, *P* = 0.001

Some significant associations were found between specific impacts and maternal background. For parity, multiparous mothers felt more negatively affected by not having visitors at home (*t*(1120) = 2.149, *P* = 0.03). For education, those without a university‐level education felt more negatively affected by not being able to have family members (*t*(1120) = 3.550, *P* = 0.000), have other visitors at home (*t*(1120) = 2.554, *P* = 0.011), not being able to attend breastfeeding support groups (*t*(1120) = 2.456, *P* = 0.014) and closure of baby clinics (*t*(1120) = −2.971, *P* = 0.003). For ethnicity, women from White backgrounds felt more negatively affected by not being able to get out to shops, baby groups and so on (*t*(1120) = −2.208, *P* = 0.02).

Participants were also asked whether they felt that lockdown led to them having more or less time to focus on breastfeeding. Those who were still breastfeeding were significantly more likely to perceive they had more time (*χ* ^2^ = 202.902, *P* = 0.000). Overall, 68.7% of those still breastfeeding felt they had much more or a little more time to focus on breastfeeding compared with 25.7% of those no longer breastfeeding. No significant associations were seen between time and maternal demographic background.

### 3.7 Impact of individual circumstances upon lockdown experience

Participants were asked a series of questions about their living arrangements during the lockdown: whether they had high‐speed Wi‐Fi, a private garden, lived in a ground floor house/flat (all yes/no) and whether they felt they had enough space in their home for everyone, lived near green space, could get out for regular walks and felt financially secure (5‐point Likert scale strongly agree to strongly disagree). For the Likert scale questions, participants were coded into yes (*strongly agree* and *agree*) versus no (*neutral*, *disagree* and *strongly disagree*).

Using a logistic regression model, these factors, alongside maternal education, were considered as predictors of continued breastfeeding (yes/no at the time of the survey). Significant factors for breastfeeding at the time of the survey included university‐level education, having high‐speed Wi‐Fi, living in a house/ground floor flat and having access to a private garden (Table 5).

**TABLE 5:** Living circumstances as predictors of continued breastfeeding and breastfeeding experience

	Predictor	*B*	SE	Wald	*df*	Sig.	Exp(*B*)
Breastfeeding or not	Education	0.796	0.157	25.522	1	0.000	2.216
	Wi‐Fi	2.576	0.423	37.031	1	0.000	13.144
	Ground floor	0.785	0.284	7.623	1	0.006	0.456
	Private garden	0.868	0.233	13.838	1	0.000	0.420
	Local walks	0.205	0.207	0.978	1	0.061	0.721
	Space in home	0.205	0.207	0.978	1	0.323	0.815
	Green space area	0.234	0.211	1.299	1	0.268	0.792
	Financial worries	4.13	0.557	0.548	1	0.459	1.511
Positive or negative lockdown breastfeeding experience	Education	0.319	0.121	6.872	1	0.009	0.727
	Wi‐Fi	0.940	0.245	14.664	1	0.000	0.391
	Ground floor	1.253	0.252	24.729	1	0.000	3.500
	Private garden	0.584	0.205	8.804	1	0.004	0.558
	Local walks	0.438	0.140	9.754	1	0.002	1.550
	Space in home	0.210	0.169	1.543	1	0.214	1.234
	Green space area	0.064	0.169	0.145	1	0.703	0.938
	Financial worries	0.391	0.396	0.972	1	0.324	1.478

Individual circumstances were also associated with the perceived impact of the lockdown upon breastfeeding experience. Participants were grouped into positive versus neutral/negative. Any participants who had responded ‘not sure’ were excluded from the analysis. A logistic regression model found significant associations between a more positive experience and university‐level maternal education, high‐speed Wi‐Fi, living in a house/ground floor flat, having a private garden and living in an area where it was easy to get out for walks/fresh air (Table 5).

Participants were asked to further reflect on how they felt the lockdown had affected their breastfeeding experience. Thematic analysis identified three broad overarching categories: those who felt they had a more positive breastfeeding experience because of the lockdown, a more negative one or were not affected at all. Those in the third category tended to have a baby over 6 months, and breastfeeding was established alongside solid foods. These mothers did not generally tend to need support—breastfeeding was straightforward, or they had established friendship groups if they had a question or concern.

I needed more support in the early days but thankfully he's older now and I have not had any issues since lockdown started that needed support.

I was Lucky to have a whats app group of breastfeeding mothers—that I had met in a baby group pre lockdown. If I had a younger baby and did not have this I would have struggled.

In terms of the other two larger groups, a number of subthemes arose. Notably, often a similar situation or issue could be perceived as either positive or negative depending on the individual's wider situation, needs and preferences.

#### 3.7.1 Positive impact

Six subthemes were identified under the category of lockdown having a positive impact. These included more time to focus, fewer visitors, more privacy, increased responsive feeding, greater partner support and a delay of return to work outside the home.

##### More time to focus

Many participants talked about a slower pace of life and nowhere to go or need to be having a positive impact on how much time they could spend focusing on feeding their baby. This was particularly helpful for some mothers who were struggling with issues such as latch. They believed that if they had been pressured to be out and about meeting people, they would still have been struggling or in pain and would have stopped breastfeeding.

I've found breastfeeding quite difficult due to problems with latching, nipple tears/bleeding and lots of pain. Being able to stay home and concentrate on getting the feeding right is the only reason I persisted. If I'd had lots of visitors or pressure to meet other mums/family/friends, I'm not sure I would have managed as I've only just got the hang of feeding 5 weeks in!

##### Fewer visitors

For some mothers, fewer visitors meant that they were more relaxed and had more time to focus on their baby and their own recovery rather than hosting a stream of people wanting to see their baby. For others, it meant fewer unwanted comments. This helped get breastfeeding off to a much better start.

I was inundated with visitors with my first child and often could not feed responsively due to their discomfort with feeding or them wanting to comfort my daughter when she was upset. She had poor growth and I felt enormous pressure from my in laws in particular to supplement with formula. With my second child, there is none of that pressure and I can really see an enormous difference both is his feeding and in my mental health.

##### More privacy

Related closely to the previous factor was the enhanced privacy mothers had. A common reason for stopping breastfeeding sooner than planned is embarrassment about feeding in front of others. Mothers who felt this way reported feeling more confident being at home and not having to feed out and about, feeling they would not have breastfed so long in public or in front of visitors. Some sat around topless, having lots of skin to skin contact. This enabled them to practice and gain confidence in latching their baby on, discretely, if desired.

Not being able to go out has allowed me to gain more confidence in bf. I still do not feel confident enough to feed in public and feel I need support with positioning to be able to do this. Not having lots of visitors also has allowed me to be able to feed how it works for us without having to worry about people coming round.

##### Increased responsive feeding

Another common experience was that the additional time and lack of pressure meant that it was easier to feed responsively, that is, responding to infant cues of hunger and satiety rather than following a mother led routine (WHO, 2003). Mothers reported feeding babies more often, to less of a routine because they did not need to plan around things like school runs and spotting earlier feeding cues. This impacted positively on perceptions of milk supply and played out in increased early weight gain.

During lock down I have had more time to focus on feeding my baby on demand and not feel rushed because I need to be anywhere.

##### Greater partner support

Depending on the working situation, some participants reported that their partner was at home for longer after the birth. Some were furloughed and had much more time to support breastfeeding and maternal recovery from both an emotional and physical perspective. Others were working from home but were still more present than they would have been if out at work all day. This shared care was felt to increase bonds between partner and baby and strengthened the new parent relationship.

My partner has been furloughed so he is here everyday with us, he can help with nappy changes, looking after our baby and letting me sleep when I need to, basically everything I'm addition to enjoying so many special moments together seeing our baby develop, having 2 of us here all the time means there's much more time for me to focus on breastfeeding our baby.

##### Delayed return to work

For mothers with a slightly older baby, a number found that their return to work coincided with the lockdown. This meant that some were furloughed or were working from home, which meant that they did not have to put their baby in childcare. This translated into more contact with their baby, more feeds and less need to express, which meant some babies got more breast milk instead of formula. It also helped mothers feel more relaxed.

I'm due back at work next week but I do not actually know what this will entail although I am unlikely to have to go in. This has meant an ease in the anxiety of continuing to breastfeed as I will still be at home after maternity leave has ended so I do not need to worry about childcare provider and expressing for bottles etc.

#### 3.7.2 Negative impact

Unfortunately, many participants described a more negative impact of lockdown upon their breastfeeding experience. Six subthemes were identified: a lack of face‐to‐face support, a lack of social support, stress of caring for other children, intense focus on breastfeeding, no experience of feeding in public and work concerns.

##### A lack of face‐to‐face support

The most common disadvantage of the lockdown was a lack of face‐to‐face breastfeeding support when mothers had difficulties. Some reported having to describe issues over the phone or from across a room, feeling that their health professional did not want to come near them. For issues such as latch, mothers really missed having someone who could look at what was happening up close and support them to make small changes.

Newborn lost a lot of weight due to tongue tie and bad latch. Breast feeding class cancelled due to COVID. Husband not permitted in hospital when breast feeding advice was given and I was recovering from giving birth so struggled to take in information. When midwife identified low weight, we were put on a feeding plan with formula and I was advised I may not be able to breast feed. I expressed a lot to ensure I could build up my supply and had very sore nipples. After contacting 111 we thought I had thrush and I was given cream. Turns out I had bad positioning which was identified via video call two weeks post birth. Face‐to‐face support e.g. somebody physically helping you to position and latch your baby is far more effective than a zoom video call on a mobile device.

Others, particularly first‐time mothers, did not realise until it was too late that support was available. They assumed that they would be provided with support, and if not contacted, then it must not be available:

I have not seen or heard of a health visitor even though my baby has missed at least on health visitor check. I did not realise there is any support out there still.

A particular issue appeared to be a lack of specialist support when it comes to diagnosing and dividing tongue ties, meaning that women were left in pain or decided to stop prematurely:

Due to having no support since my baby was 2 weeks old, I've had to adapt our feeding to allow for her possible tongue tie. I cannot even get a diagnosis due to current situation. Its been a hard slog and I've been in immense pain.

This lack of support meant that some women were either having to express (due to pain or poor latch), give formula when they did not want to or stopped feeding earlier than planned.

We are unable to have face‐to‐face support to help me to feed my baby who is struggling to gain weight due to possible tongue tie which is unable to be treated. Because of the pressure to have him gain weight or be admitted to hospital and having little support with improving his latch and expressing milk I have had to top up with formula which is something I have not wanted to do and did not need to do with my first child.

##### A lack of social and emotional support

Many participants talked about missing meeting other breastfeeding mothers and socialising in baby groups or out with friends. Sometimes, this was about asking others questions or seeking reassurance, but often, it was just about connection and feelings of community. Many talked about the isolation they felt, which was impacting their well‐being and mental health.

My previous two children had tongue ties and we never successful established breastfeeding. I was so determined this time and hoping to attend groups and have access to face‐to‐face support. The lack of support has really upset me. Sad, sad situation and not what I hoped it would be like at all. I researched so much before baby was born and cannot access any of the support I thought I could.

Additionally, in contrast to those who felt shielded from negative family interference, others felt isolated and missed the emotional support they would receive from caring and supportive relatives.

My mother is wonderful and a huge supporter of breastfeeding. I was really looking forward to her coming to visit after my baby was here. She cannot come and whilst we can video message it's just not the same as having your mum close by. I feel I need her, not just to help but emotionally and I'm struggling without this support. It makes everything feel so much harder.

##### Stress of trying to juggle caring for older children without family support

In contrast to participants who felt that being home with other children reduced pressures, others found that having older children home, needing to homeschool and not being able to get out and about really threatened their ability to establish breastfeeding. This was especially true for those with partners working outside the home still and was exacerbated by not being able to rely on family support.

I have much less time to focus on breastfeeding now since lockdown. My partner is a police officer he is at work from the crack of dawn until late at night, sometimes staying at work for 24 hrs or more depending on how situations unfold. I am at home with an energetic 5‐year‐old who would normally be in school. I do not have time to express in between feeds or sometimes breastfeed at all because I feel I need to meet my daughters demands and run the house and basically be a single parent most of the time. Before lockdown I was able to have my mum and sisters come and stay and help out. I could also have friends to help me or my older daughter could go for play dates to allow me to focus on the baby.

##### Intense focus on breastfeeding

Again, in contrast to mothers who relished the additional time to focus on feeding, some felt that the lack of any other activity or time out of the house meant that all they did was feed, feeling overwhelmed by the experience. This made them really dislike breastfeeding, feeling they desperately needed a break and something else to do and focus on.

My focus on breastfeeding was intense due to being in lockdown and almost all consuming. This is turn led me to dislike it as it felt myday was centred around it.

##### No experience of breastfeeding in public

This factor is a further example of contrasting experiences based on wider factors. Whereas some mothers preferred not having to feed in public, getting chance to practice at home first, others worried that they were missing out on this experience, which left them feeling awkward and unskilled. They worried about what would happen in the future once the lockdown was lifted.

Being able to go to clinics/clubs and get used to feeding in public with other like‐minded mum's or getting help with positioning in different situations is what is missing. This overall makes me feel like I would stop breast feeding sooner than planned as once not confined to my home I do not know how to do it with confidence.

##### Work pressures

For mothers of older children, some had returned to work and were expressing during the day to feed their babies. This was predominantly related to mothers who were key workers, particularly health professionals, meaning their experience was very different from those working at home. Here, mothers discussed how very busy schedules, stress and lots of personal protective equipment (PPE) meant they were hot and dehydrated and had little time to express, meaning they had less milk or were feeling very engorged.

Work has impacted my breastfeeding journey as I feel I am pumping less milk at the moment. And I think this is because of dehydration wearing PPE and so not sipping water all day long.

To bring these findings together, it is clear that many breastfeeding mothers in the United Kingdom have had a very divided experience when it comes to feeding their baby during the pandemic. For some, their experience has been so much easier, yet for others, numerous barriers have been placed in their way.

## 4 DISCUSSION

This study explored women's experiences of breastfeeding during the COVID‐19 pandemic, specifically in relation to how lockdown measures affected their infant feeding decisions. It clearly showed two very different experiences emerging, one where women felt more able to establish and maintain breastfeeding and one where women felt lockdown created and exacerbated issues. Somewhat unsurprisingly, those who stopped breastfeeding during lockdown had a more difficult experience, with many blaming it for having to stop breastfeeding before they were ready. Overall, the findings have important considerations for those working in breastfeeding support and policy and should be used to reflect on provision in any future similar situations (Renfrew, Cheyne, Dykes, et al., 2020).

Taking the issue of misinformation first, women receiving incorrect information about the safety of COVID‐19 and breastfeeding, being told they cannot breastfeed their baby if they had symptoms, or being separated from them after birth is a major concern. At the start of the pandemic, mothers and babies were routinely separated in China, Malaysia, the Philippines, Indonesia and more, with breast milk substitutes sometimes recommended (Tomori, Gribble, Palmquist, Ververs, & Gross, 2020). Likewise, the American Academy of Pediatrics recommended separating mother and baby in case of suspected infection (Bartick, 2020)—guidance that has now thankfully changed (AAP, 2020). Although a number of organisations in the United Kingdom were quick to state that breastfeeding was safe and to be encouraged (UNICEF UK, 2020), professionals and parents alike may still have been exposed to these messages. This was not helped by the spread of misinformation on social media, something that was rife across different impacts upon health during the pandemic (Singh et al., 2020). If a lockdown occurs again, it is vital that policy makers ensure rapid, clear and visible support for continued breastfeeding both across health care professionals and social media messaging.

Separation of mother and baby goes against everything we know about supporting breastfeeding initiation (Pérez‐Escamilla et al., 2016; Renfrew, Cheyne, Craig, et al., 2020) and promoting stable newborn behaviour (Ahn, Ko, Kim, Lee, & Shin, 2008). Although the overall percentage of women being told breastfeeding was not safe or being prevented from being with their newborn baby was low in our study, it led to breastfeeding cessation or fears around safety, even amongst some mothers of older infants where breastfeeding was established. Over four in five women prevented from visiting their baby in the NICU had stopped breastfeeding by the time they completed this study. That statistic is unsurprising; it has long been recognised that keeping mother and baby together particularly when her baby is premature and in the NICU plays a vital in breastfeeding success (Cuttini et al., 2019; Renfrew et al., 2010). Meanwhile, new mothers can already be anxious about the safety of their breast milk (Kronborg, Harder, & Hall, 2015) even before they receive such messages from trusted health professionals.

It is likely that some health professionals or hospitals will have been erring on the side of caution, particularly at the start of an epidemic. If a baby had become critically ill or died because of COVID‐19 infection transferred from a mother when it was preventable, the consequences would be severe. This was not helped by confusion amongst different sources of health information; misinformation and fear may have spread before this stage. We also know that not all health professionals are supportive of breastfeeding or believe that if any perceived risk presents itself, it should not be encouraged (Watkins & Dodgson, 2010).

However, we now know that mother to infant transmission during pregnancy or after the birth appears uncommon, with infants having low rates of infection and reduced risk of severe disease and complications (Walker et al., 2020). Overwhelmingly, reports on the presence of SARS‐CoV‐2 in human milk samples have been negative (Smith et al., 2020). One case study from Australia found that when a symptomatic mother took suitable precautions (e.g., mask wearing and handwashing), her infant did not contract the illness (Lowe & Bopp, 2020), and these precautions are emphasised by WHO (2020b). Although a small number of case reports identified low numbers of viral fragments in samples of human milk from symptomatic mothers (Groß et al., 2020), a systematic review by the WHO highlighted that no report had demonstrated the presence of intact viral particles, and viral infectivity cell culture assays had not yet been performed (WHO, 2020b). We also know that from looking at similar outbreaks such as the 2003 SARS‐CoV virus, there was no evidence of transmission into breast milk (CDC, 2020).

Conversely, we know that breast milk passes immune protection to the infant if a mother comes into contact with an infection (Goldman, 1993). We also know that in the previous SARS‐CoV epidemic, women who were infected were found to produce antibodies in their milk (Robertson et al., 2004). Given the likelihood that if a mother is exposed to COVID‐19, she will expose her baby before she develops symptoms herself, breast milk is likely to offer protection to infants. The WHO has stated that mother and baby should not be separated because of COVID‐19 unless absolutely essential (WHO EURO, 2020). However, clearly, further widespread training may be necessary to ensure unnecessary separation does not continue now or in future emergencies. Simply ensuring that the UNICEF UK Baby Friendly Initiative Standards that promote keeping mother and baby together, responsive care and supporting informed feeding decisions (BFI, 2020) are upheld across facilities would go a long way to supporting this.

Moreover, what is often ignored in discussions around breastfeeding safety are the consequences of not breastfeeding; breastfeeding offers long‐term protection for maternal and infant health (Acta Pediatrica, 2015; Victora et al., 2016). Additionally, breastfeeding protects maternal well‐being physically by reducing the risk of inflammation, enhancing sleep and moderating stress (Kendall‐Tackett, 2007) and psychologically (Brown, 2019). When mothers meet their own breastfeeding goals, their mental health is protected, but when they cannot, particularly if they experience complications, their risk of depression, grief and trauma increases. When weighing up the potential risks of breastfeeding, the whole picture must be taken into consideration.

This takes us to the issue of the number of women stopping breastfeeding during the lockdown, many of whom did not access support and felt that changes to support directly impacted their decision to stop breastfeeding. Mothers who gave birth during the pandemic, as against before it, were more likely to have stopped breastfeeding at the time of the survey. This will likely be confounded by these mothers having a younger infant and therefore being more likely to encounter breastfeeding difficulties and consequently being at greater risk of stopping breastfeeding (McAndrew et al., 2012), although when mothers of older infants did encounter difficulties, they too felt they were affected by a lack of face‐to‐face support. However, mothers who gave birth in lockdown reported feeling more negatively affected by the pandemic and lockdown both in terms of feeling they did not get enough support and that limitations on social contact prevented them from accessing support services and social opportunities. It is likely that mothers of an older infant may either not rely on such opportunities so heavily, or have already made connections, and raises critical points for the importance of focussed support in the early weeks of breastfeeding.

We know that professional and peer support plays a vital role in breastfeeding success (Ingram et al., 2020; McFadden et al., 2017; Thomson, Crossland, & Dykes, 2012; Trickey et al., 2018), but when exploring qualitative responses, it was clear that women who stopped breastfeeding were affected by a lack of practical, emotional and peer support. Women, particularly in the early weeks of breastfeeding where care needs are highest, were affected by not being able to see health professionals face to face or when appointments did occur feeling uncomfortable. Services such as tongue‐tie division were unavailable through usual NHS routes, and few women appeared to know about private options. Many of these women directly blamed the pandemic and lockdown on being unable to continue. Very few felt ready to do so, and most had planned to breastfeed for longer—an experience we know is related to increased risk of postnatal depression (Brown, Rance, & Bennett, 2016).

It has also been recognised that the pandemic has had an impact on morbidity and mortality rates outside of COVID‐19 infection, for example, through delayed access to services, cancellations and avoiding hospitals (ONS, 2020). It is important that policy makers now track the impact upon population breastfeeding duration and subsequent physical and mental health outcomes. As noted previously, not breastfeeding has long‐term health consequences at the population level for both women and children, the impact of which may not be seen for some time. What is more imminent is the need for support for women who have not been able to meet their breastfeeding goals or who have struggled through this period in pain because of a lack of support (Brown, 2018). Evidence is also emerging that rates of perinatal depression increased during COVID‐19 lockdowns in China (Wu et al., 2020) and Canada (Davenport, Meyer, Meah, Strynadka, & Khurana, 2020). This is unsurprising; we know that social isolation, lack of professional support, health worries and financial difficulties—of which COVID‐19 created the perfect storm—increase the risk of perinatal depression (Redshaw & Henderson, 2013). Policy makers must ensure families receive the holistic support they need from now on (Institute of Health Visiting, 2020).

Finally, it is clear that women's experiences of COVID‐19 and breastfeeding differ significantly according to their experience of the pandemic and lockdown. From a positive perspective, 41.8% of mothers actually reported that the pandemic had a positive impact on their infant feeding experiences. It appeared that it forced or encouraged some mothers into situations where they were able to do a lot of things that we know support breastfeeding well: increased time to get breastfeeding established, fewer interruptions, more time with supportive partners and protection from unwanted opinions (Brown, 2016). Effectively, these women's experiences emulate that seen in many cultures where postnatal recovery and care are prioritised through rest, food and care (Dennis et al., 2007).

What is clear from the findings, however, is that mothers who found the experience more positive were more privileged in their living circumstances. They had more space in their homes, access to gardens and green space for exercise, fast Wi‐Fi connections and fewer financial difficulties. Breastfeeding and, more broadly, caring for their infant were supported by the environment in which they lived. Although stress itself does not impact milk production, it can inhibit the milk ejection reflex, making breastfeeding more difficult (Dewey, 2001). It is likely that those living without these advantages may find breastfeeding a very different experience. Reflecting back on known influences on postnatal depression such as isolation, money worries and stress, it is likely women in these experiences find caring for a baby more difficult, and breastfeeding is a large part of that caring experience. This is before we consider the increased occurrence of domestic violence and relationship difficulties during lockdown (Usher, Bhullar, Durkin, Gyamfi, & Jackson, 2020).

The concern that arises is the divide in breastfeeding experience that the pandemic has created or more accurately widened. We know that mothers with a lower education (e.g., school leavers ≤ 18 years) and income level are more likely to stop breastfeeding in the early weeks, experiencing more difficulties and less support (Brown, Raynor, & Lee, 2011; McAndrew et al., 2012). In our study, mothers without a university‐level education had fewer advantages in their living circumstances and reported a more negative impact of lockdown upon their breastfeeding experience. However, living circumstances impacted mothers independently of education level, suggesting a direct impact of more disadvantaged circumstances.

Notably, mothers from BAME populations were also more likely to have stopped breastfeeding and have found the lockdown experience more negative. Given data that BAME mothers in the United Kingdom typically initiate and continue breastfeeding at a higher rate than White women (McAndrew et al., 2012), this is especially concerning. Although women from BAME backgrounds perceived they had less practical support during the lockdown and were more likely to attribute breastfeeding cessation to a lack of professional support, there were no other differences in feeding experiences (e.g., reactions to lockdown), suggesting further external factors may be at play. Evidence is mounting that racial disparities in access to care and increasing racism in response to the pandemic are putting BAME communities at increased risk of negative outcomes from the pandemic (Coates, 2020; Lacobucci, 2020). It has already been emphasised that greater perinatal support is needed for BAME women (Renfrew, Cheyne, Craig, et al., 2020), but our data further show that this appears to be extending to breastfeeding experiences and urgently needs greater investigation and support put in place.

The question arises as to how we can make a difference moving forward. It is unlikely that face‐to‐face breastfeeding support will return to prepandemic levels in the near future, with some families needing to shield for longer. Online and telephone support is likely to remain as a core support mechanism for some time. It is critical that we ensure positive and accurate messaging and support services reach all mothers and that we overcome the barriers some are facing. Although some mothers felt supported by the online delivery of support, they were potentially already better informed and connected to sources of support in the first place or, as noted above, had the resources to access such services easily. Others struggled, finding online support impersonal, inaccurate or difficult to access.

How do we ensure more equitable and accurate delivery of telemedicine for breastfeeding support? Challenges of using this approach to provide patient care have already been identified for COVID‐19 (alongside many benefits). Connectivity and missing nuances due to a lack of in‐person care are central to this (Calton, Abedini, & Fratkin, 2020), alongside broader issues of needing to train staff, technology availability and education level (Scott Kruse et al., 2018). It has been suggested that telephone conversations may replace video calls (Calton et al., 2020), but the accuracy of this for supporting common breastfeeding issues are unclear. Although telemedicine support can improve breastfeeding duration and exclusivity (dos Santos, Borges, & Zocche, 2019; Kapinos et al., 2019), it is well recognised that women do not simply value practical breastfeeding support—they value the emotional care of professionals and peers too (Schmied, Beake, Sheehan, McCourt, & Dykes, 2011), and that is more likely to occur with face‐to‐face contact within an established community setting (Demirci, Kotzias, Bogen, Ray, & Uscher‐Pines, 2019). However, research has not as yet explored how to efficiently provide such support within the context of a global pandemic. Further research should explore mothers' needs during such a time.

The research does have limitations. Using online research data collection methods is an increasingly popular approach and one that was necessary during the pandemic lockdown. However, it would likely have excluded participants from the most deprived groups who could not access the internet or were managing significant stress, which our data show are key indicators of early breastfeeding cessation. It is possible that mothers with the most positive or negative experiences were more likely to complete the survey, and potentially, a larger group of mothers who were less affected did not complete it. Using infant feeding organisations to aid dissemination may have exacerbated this, but it increased visibility of the research request.

Additionally, like many similar surveys, it was also weighted towards mothers with a higher level of education and age and from disproportionately White participants compared with the general population. We had to group all mothers from BAME populations together to have sufficient sample sizes for statistical analyses but realise this is a reductionist approach, which ignores differences in experiences between different population groups. Our data, however, serve to further add weight that specific, larger research must be conducted to better understand the experiences of BAME populations both in relation to COVID‐19 and infant feeding. Overall, given the challenges of lockdown and the importance of collecting data during this period, this method of data collection proved a useful way of collecting a large sample of responses, which did contain sufficient numbers to explore experiences by different demographic groups. Caution should be given to generalisation, but the findings offer suggestions to where further research and resources should be directed.

Limitations aside, the findings are important in highlighting the impact of the pandemic upon infant feeding experiences in the United Kingdom. Although larger population‐scale data are needed, our findings suggest that the impact of COVID‐19 and lockdown upon breastfeeding rates may be very mixed. Whereas some mothers have been enabled to breastfeed for longer, others have felt forced to stop before they are ready. What the overall impact of this upon national breastfeeding rates will be is yet to be seen. What is clear, however, is that the pandemic disproportionately affected some mothers, particularly those from more deprived communities, making their infant feeding experiences more difficult. We cannot change what has already occurred, but we can offer further support to mothers who have experienced it and make sure that in future similar situations, all families continue to receive the support they need.

## CONFLICTS OF INTEREST

The authors declare that they have no conflicts of interest.

## CONTRIBUTIONS

AB and NS were both responsible for study design, data analysis, draft writing and critical revisions.
